# Pathogenic Viruses Commonly Present in the Oral Cavity and Relevant Antiviral Compounds Derived from Natural Products

**DOI:** 10.3390/medicines5040120

**Published:** 2018-11-12

**Authors:** Daisuke Asai, Hideki Nakashima

**Affiliations:** Department of Microbiology, St. Marianna University School of Medicine, Kawasaki 216-8511, Japan

**Keywords:** anti-human immunodeficiency virus (HIV), antiviral, natural product, human virus

## Abstract

Many viruses, such as human herpesviruses, may be present in the human oral cavity, but most are usually asymptomatic. However, if individuals become immunocompromised by age, illness, or as a side effect of therapy, these dormant viruses can be activated and produce a variety of pathological changes in the oral mucosa. Unfortunately, available treatments for viral infectious diseases are limited, because (1) there are diseases for which no treatment is available; (2) drug-resistant strains of virus may appear; (3) incomplete eradication of virus may lead to recurrence. Rational design strategies are widely used to optimize the potency and selectivity of drug candidates, but discovery of leads for new antiviral agents, especially leads with novel structures, still relies mostly on large-scale screening programs, and many hits are found among natural products, such as extracts of marine sponges, sea algae, plants, and arthropods. Here, we review representative viruses found in the human oral cavity and their effects, together with relevant antiviral compounds derived from natural products. We also highlight some recent emerging pharmaceutical technologies with potential to deliver antivirals more effectively for disease prevention and therapy.

## 1. Introduction

The human oral cavity is home to a rich microbial flora, including bacteria, fungi, and viruses. Oral tissues are constantly exposed to these microbes, which form a complex ecological community that influences oral and systemic health [1]. Discussion of the microbiological aspects of oral disease traditionally focuses on bacteria and fungi, but viruses are attracting increasing attention as pathogens. Viruses are generally more difficult to detect among pathogenic microbes, at least with traditional methods such as in vitro cultivation; however, the development of sophisticated molecular tools, including monoclonal antibodies and viral genome sequencing, have greatly advanced the field of virology over the past decade or so. A number of viruses have been found in the oral cavity, of which many are thought to be involved in the development of various types of oral ulcers, oral tumors, classical oral infectious diseases, and periodontitis. For example, herpes simplex virus 1 (HSV-1) causes gingivostomatitis, and the virus can subsequently enter a dormant state in the trigeminal ganglion. Blood-borne viruses such as human immunodeficiency virus (HIV) can enter the mouth via gingival crevicular fluid, and viruses causing upper respiratory tract infections are also found in the mouth [2]. Similarly, the mumps virus is known to infect the salivary glands and can be found in saliva of affected individuals. Human papillomavirus (HPV) is responsible for several oral conditions, including papilloma, condylomas, and focal epithelial hyperplasia, and has also been implicated in head and neck squamous cell carcinoma.

The field of antiviral research acquired new urgency since the 1980s, owing to the global spread of HIV, which causes acquired immune deficiency syndrome (AIDS). HIV is a member of the RNA retroviruses, which contain a reverse transcriptase (RT) enzyme that transcribes RNA into DNA in infected cells, leading to integration of the retroviral genomic information into chromosomal DNA of the host cell. Antiretroviral drugs (ARVs) often target RT, but are always given in combination with other ARVs for antiretroviral therapy (ART) in order to increase efficacy and reduce the development of resistance. The anti-HIV agent zidovudine (AZT) is a representative ARV, and was the first to be approved in the United States in 1986; however, recently, many new classes of drugs have been introduced [3], together with new formulations such as long-acting depot-type anti-HIV drugs and easy-to-use gel formulations for preventing rectal HIV infection [4].

The enormous research effort directed at the treatment of HIV has led to important advances in basic science and many therapeutic breakthroughs, including the development of inhibitors targeting a range of human viruses. Nevertheless, large-scale screening of natural products, such as extracts of marine sponges, sea algae, plants, and arthropods, remains an important source of leads for new antiviral agents, whose potency and selectivity can then be optimized with the aid of rational design strategies, including computational approaches. Thus, the aim of this review is to provide a personal viewpoint on natural-product-derived antiviral agents that are available for the treatment of pathogenic viruses, especially HIV, that may cause symptoms in the oral cavity, considered from a historic perspective. As major candidates for viruses involved in oral diseases, we focus on (1) HSV-1 and HSV-2; (2) Epstein–Barr virus (EBV); (3) Kaposi sarcoma-associated herpesvirus (KSHV); (4) human papilloma virus (HPV); and (5) HIV.

## 2. Viruses Associated with Oral Diseases of Humans

Members of the human herpesvirus (HHV) family cause common primary viral infections of the oral mucous membrane, and may also play a role in periodontitis. There are eight members of the HHV family, which are among the largest and most complex human viruses. We focus here on HSV-1, HSV-2, EBV, and KSHV; the others are varicella-zoster virus (VZV), human cytomegalovirus (HCMV), HHV-6, and HHV-7 (Table 1). HPV is a diverse family of viruses, which can cause a variety of pathologies, including oral squamous cell carcinoma, and chronic infection of the skin or mucosal epithelium. Furthermore, the retrovirus HIV causes a decline in immunocompetence, which can lead to various cutaneous manifestations [5]. In this section, we present a brief overview of these viruses.

### 2.1. HSV-1 and HSV-2

HSVs are relatively large, enveloped, double-stranded DNA viruses with icosahedral symmetry. Infections by HSV-1 are referred to as upper body infections to distinguish them from the genital infection caused by HSV-2. The formal designations of these viruses are human herpesvirus 1 (HHV-1) and 2 (HHV-2). Oral herpes is a viral infection mainly around the mouth and lips caused by herpes simplex viruses. HSV-1 causes painful sores on the upper and lower lips, gums, tongue, roof of the mouth, inside the cheeks or nose, and sometimes on the face, chin, and neck. In addition, HSV-1 can cause symptoms such as swollen lymph nodes, fever, and muscle aches. HSV-2 is primarily associated with the genitals and most often causes genital herpes. However, it may spread to the mouth during oral sex, causing oral herpes. HSVs characteristically establish latent infection in sensory nerve ganglia, and, in this case, signs appear only when the virus is reactivated. Following recovery from primary oropharyngeal infection, the individual retains HSV DNA in the trigeminal ganglion, but the virus becomes dormant. Infected individuals have at least a 50% chance of suffering sporadic recurrent attacks of herpes labialis (otherwise known as facial herpes, herpes simplex, fever blisters, or cold sores) from time to time throughout the remainder of their life.

At present, the drug of choice for the treatment of HSV infections is acyclovir (ACV; 9-(2-hydroxyethoxymethyl)guanine). Its unique advantage over earlier nucleoside derivatives is that HSV-encoded thymidine kinase (TK), which has broader specificity than cellular TK, phosphorylates ACV to ACV-monophosphate (ACV-P). A cellular guanosine monophosphate (GMP) kinase then completes the phosphorylation to generate the active agent, ACV-triphosphate (ACV-PPP). ACV-PPP acts as both an inhibitor and a substrate of the viral enzyme, competing with guanosine triphosphate (GTP) for incorporation into DNA; this results in chain termination, because ACV lacks the 3’-hydroxyl group required for chain elongation. Before ACV was introduced, vidarabine (Ara-A) which was the first nucleoside analog antiviral to be systemically administered, was used. Ara-A is also sequentially phosphorylated by kinases to afford the triphosphate ara-ATP, which competitively inhibits deoxyATP (dATP). However, the mechanism of Ara-A is different in that all three phosphorylations are mediated by host adenosine kinases, resulting in a lack of specificity. Indeed, Ara-A is more toxic and less metabolically stable than ACV, although it is still employed against ACV-resistant strains. In addition, valaciclovir (VACV), an ester of ACV, is a prodrug that has greater oral bioavailability than ACV. VACV provides high plasma levels of the parent compound and offers greater efficacy, as well as decreased dosing frequency. Additionally, guanosine analog famciclovir (FCV) is another choice for the treatment of HSV infections.

### 2.2. EBV

EBV, formally human herpesvirus 4 (HHV-4), has a small DNA genome, and its main host cells are B lymphocytes and epithelial cells. EBV replicates in epithelial cells of the nasopharynx and salivary glands, especially the parotid, lysing them and releasing infectious virions into saliva. EBV attaches to B lymphocytes via binding of the viral glycoprotein gp350/EBV to CD21 receptors on the lymphocyte. The major characteristic of infected B lymphocytes is transformation. When this occurs, only a small amount of the viral DNA integrates into the host chromosome, and most of the viral DNA stays in a separate circular episome form. The B lymphocytes infiltrating the lymphatic tissue of the infected oropharyngeal mucosa may, in turn, become infected, but are generally not permissive for virus production. EBV is transmitted only after repeated contact with infected individuals. It can manifest in the oral cavity and/or head and neck region, e.g., as Burkitt’s lymphoma (BL), mononucleosis, or oral hairy leukoplakia (OHL), and the prevalence and disease severity are increased in individuals co-infected with HIV. Mononucleosis is common, irrespective of HIV infection status, and is associated with a primary EBV infection during adolescence and young adulthood. OHL is an oral mucosal lesion that is associated with EBV infection and is often asymptomatic, commonly presenting as non-removable white patches on the lateral borders of the tongue. OHL is now an established phenomenon in a range of conditions affecting immune competence, e.g., in immunosuppressed patients with HIV infection or bone marrow transplant recipients [6]. EBV-positive Hodgkin’s and non-Hodgkin’s lymphomas may manifest in the head and neck. Nasopharyngeal carcinoma (NPC) is also a head and neck cancer associated with EBV infection. No effective anti-EBV drugs have yet been developed. EBV is sensitive to ACV in vitro, but systemic administration of this drug has little effect on clinical illness.

### 2.3. KSHV

The formal designation of human herpesvirus 8 (HHV-8) was proposed for KSHV, in keeping with the systematic nomenclature adopted for all human herpesviruses. KSHV is the causative virus of Kaposi sarcoma (KS), multicentric Castleman’s disease, and primary effusion lymphoma. Humans are thought to be the natural host for KSHV, which is primarily transmitted via saliva. Infection occurs during childhood and increases with age. KSHV is detected in endothelial and spindle cells of Kaposi sarcoma lesions, as well as in circulating endothelial cells, primary effusion lymphoma cells, B lymphocytes, macrophages, dendritic cells, oropharynx and prostatic glandular epithelium, and keratinocytes. Epidemiological studies indicate that there are four clinical variants of KS: (1) classic; (2) endemic (African); (3) iatrogenic (transplant-associated); and (4) HIV/AIDS-associated (epidemic) (HIV-KS). It is well known that KS is an AIDS-defining illness and the most frequent AIDS-associated neoplasm. HIV-KS commonly affects the oral cavity, with the oral mucosa being the initial site of clinical disease in 20% of patients [7]. ART has successfully decreased the prevalence and incidence of HIV-KS. A liposome-encapsulated form of doxorubicin (Doxil) is often used primarily for the treatment of AIDS-related KS.

### 2.4. HPV

HPV is a small, non-enveloped, double-stranded DNA virus with icosahedral symmetry. There is wide genetic diversity among HPVs, and more than 70 genotypes of HPVs have been identified so far. Some of them are associated with a variety of benign papillomatous lesions of the skin and squamous mucosa. The mechanism of malignant transformation is not fully understood and is difficult to study because HPV is difficult to grow in culture. It is thought that the viral DNA remains episomal in benign lesions, whereas it is integrated into host chromosomal DNA in malignant cells, e.g., cervical carcinoma. Oral papillomas of the conventional kind can be caused by sexually transmitted HPV types 6, 11, and 16. Common warts are most frequently caused by type 2 [8].

HPVs induce benign tumors of the epithelium in their natural host. The discovery of the role of HPV in cervical cancer has led to the widespread use of HPV vaccines for young women, but it is still uncertain whether HPV actually plays an essential role [9]. Based on their putative role in cervical carcinoma, the viruses are classified as having either high (primarily 16 and 18) or low (primarily 6 and 11) oncogenic potential. HPVs are often found in oral samples from healthy mouths, such as brush samples of mucosa, but their prevalence is typically reported to be higher in biopsies from oral lesions, including leukoplakia and/or cancers. The association with oncogenic HPVs is less obvious in the case of leukoplakia, while there are many reports of HPVs in malignant cancers. The observed prevalence in oral cancers is considerably lower than that in cervical cancers, but the case for a role of HPVs is still reasonably strong [2]. When generations who have received papillomavirus vaccines grow up, we should find out whether the prevalence of oral carcinomas declines along with the expected decline in cervical cancer. Imiquimod is used as a patient-applied cream to treat genital warts. Imiquimod is a Toll-like receptor 7 (TLR7) agonist, promoting the secretion of inflammatory cytokines. Traditional treatments consist of locally destructive techniques, such as cautery, surgical excision, and cryotherapy using liquid nitrogen.

### 2.5. HIV

**Figure 1:**
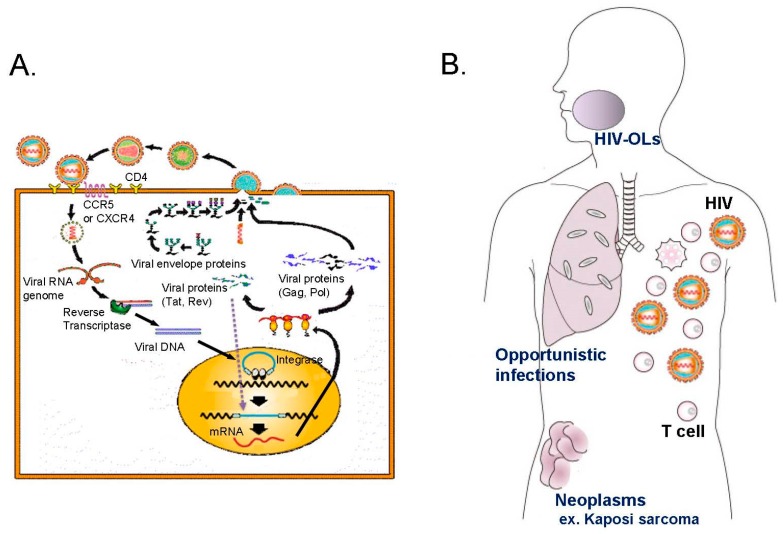

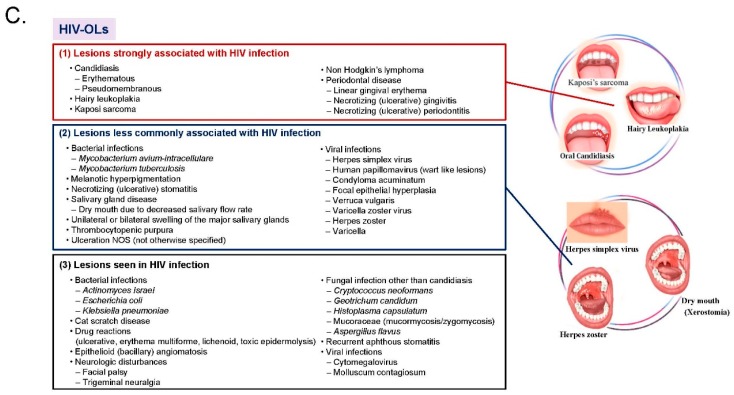
Life cycle and effects of human immunodeficiency virus (HIV). (**A**) The HIV infection of cells begins when the envelope glycoprotein of a viral particle binds to both cluster of differentiation 4 (CD4) and a co-receptor that is a member of the chemokine receptor family. Once inside the cells, the viral genome is reverse-transcribed into DNA and incorporated into the cellular genome. Viral gene transcription and viral reproduction are stimulated by signals that normally activate the host cell. Production of the virus is accompanied by cell death. (**B**) Infection with HIV induces immunosuppression, which in turn enables many kinds of bacteria, fungi, and viruses to grow in the oral cavity. HIV-infected individuals usually exhibit immune dysfunction prior to depletion of their CD4-positive T-helper cells. The progressive immune deficiency is accompanied by a wide range of opportunistic infections and neoplasms, such as candidiasis, Kaposi sarcoma, and hairy leukoplakia, in the oral cavity. (**C**) The 1993 EC-Clearinghouse classification for oral lesions associated with HIV (HIV-OLs) is still globally used, despite some controversy as to its current relevance to periodontal diseases.

HIV is an enveloped retrovirus that is transmitted through sexual contact or by contact with infected body fluids. Retroviral RT allows the virus to integrate its genetic information into the host chromosome. HIV targets CD4-positive T-helper cells, and the resulting development of immunodeficiency leads to AIDS. Viral infections are a significant cause of morbidity and mortality in immunosuppressed patients. In general, diseases or medical treatments that have cytostatic or cytotoxic effects on lymphocytes increase the risk of viral infections, and the viral infection rate depends on the nature and degree of immunosuppression. The reactivation of latent virus is the most important determinant of the type of viral infection, and this occurs most commonly in immunosuppressed patients. Skin and mucous membrane manifestations of HIV infection may result from opportunistic disorders secondary to the decline in immunocompetence caused by the infection. HIV-related oral conditions occur in a large proportion of patients, and frequently are misdiagnosed or inadequately treated. Dental expertise is necessary for appropriate management of oral manifestations of HIV infection; however, in practice, many patients do not receive adequate dental care. Common or notable HIV-related oral conditions include the following symptoms: xerostomia (dry mouth), candidiasis, OHL, periodontal diseases such as linear gingival erythema and necrotizing ulcerative periodontitis, KS, HPV-associated warts, ulcerative conditions including HSV lesions, recurrent aphthous ulcers, and neutropenic ulcers. In 1993, consensus was reached on the classification of the oral manifestations of HIV, so-called the 1993 EC-Clearinghouse classification. It classifies oral lesions associated with HIV (HIV-OLs) into three groups: (1) lesions strongly associated with HIV infection; (2) those less commonly associated with HIV infection; and (3) lesions seen in HIV infection [10]. The sequence of events associated with HIV infection, from the cellular level of infection to oral manifestations in HIV-infected patients, is illustrated in Figure 1, together with the classification of HIV-OLs.

HIV is one of the best-studied viruses and, thus, anti-HIV agents show the widest range of structural variation among antiviral agents. Since the introduction of combination therapy (ART) for patients, HIV infection has been transformed into a long-term and manageable disorder; indeed, ART can reduce plasma virus titers to below detectable levels for more than one year and slow the disease progression. The major classes of drugs used in ART regimens include entry inhibitors (EI), nucleoside reverse-transcriptase inhibitors (NRTI), non-nucleoside reverse-transcriptase inhibitors (NNRTI), protease inhibitors (PI), and integrase strand-transfer inhibitors (INSTI).

## 3. Natural Products as Antiviral Agents

### 3.1. Early History of Antivirals

**Figure 2:**
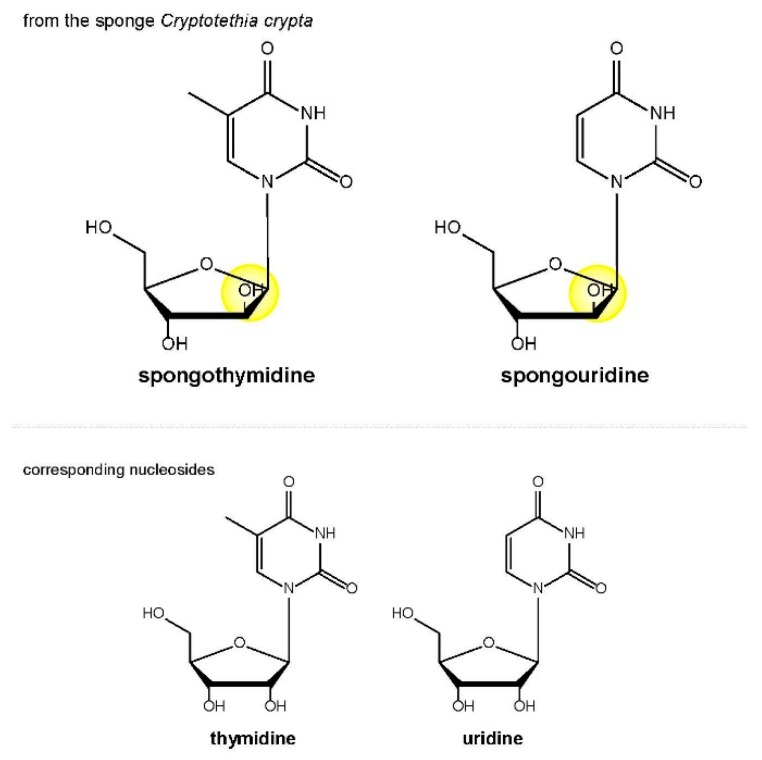
Chemical structures of arabinosyl nucleosides from the sponge *Cryptotethia crypta*, together with the corresponding human nucleosides. The key structural features are highlighted in yellow. Arabinosyl nucleosides contain arabinofuranose instead of β-d-ribofuranose.

Extracts of natural materials, such as herbs, spices, roots, tree barks, leaves, etc., have a long anecdotal, as well as proven history of use in treating human ailments. Indeed, many drugs now in clinical use have their origins in plants, marine organisms, bacteria, and fungi that were traditionally believed to have desirable pharmacological activities. However, it is difficult to isolate pure active compounds from a complex array of substances, some of which may be cytotoxic, present in natural materials. Nevertheless, the discoveries of antiviral-active nucleosides, spongothymidine and spongouridine, more than half a century ago made scientists aware of the potential value of antivirals from natural sources (Figure 2) [11].

### 3.2. Antivirals in the Latter Half of the 20th Century

**Figure 3:**
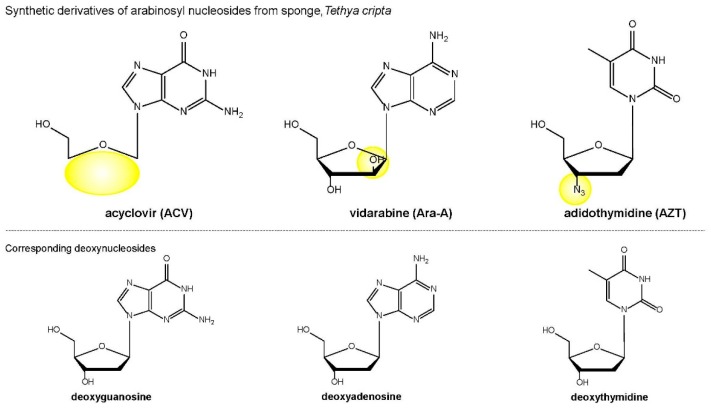
Chemical structures of nucleic acid analogs clinically used as key antivirals today, together with those of analogous competitors. Acyclovir (ACV) is used for the treatment of herpes simplex virus infections, chickenpox, and shingles. ACV inhibits viral DNA polymerase activity in a deoxyguanosine triphosphate (dGTP)-competitive manner. Vidarabine (Ara-A) is active against herpes simplex and varicella zoster viruses. Ara-A inhibits viral DNA polymerase activity in a deoxyadenosine triphosphate (dATP)-competitive manner. Azidothymidine, also called zidovudine (AZT), is the most common drug prescribed for individuals who have acquired immune deficiency syndrome (AIDS). AZT inhibits viral reverse transcriptase (RT) activity in a deoxythymidine triphosphate (dTTP)-competitive manner. The key structural features are highlighted in yellow.

Since the early work on marine-derived antivirals, thousands of novel compounds with antiviral activities have been isolated from natural sources, and some have been successfully developed for clinical use. Perhaps the most important contribution was the isolation and characterization of arabinosyl nucleosides from a Caribbean sponge (phylum Porifera) called *Tethya cripta* (Tethylidae), which provided the basis for drug design of nucleoside analogs used in medicine today. In this context, the most important antivirals that have come onto the market so far are acyclovir (ACV) [12], vidarabine (Ara-A) [13], and azidothymidine (zidovudine) (AZT) [14] (Figure 3). ACV and Ara-A are nucleic acid analogs that competitively inhibit herpes viral DNA polymerases, preventing further viral DNA synthesis. As for AZT, the active intracellular metabolite, AZT-triphosphate, is an HIV RT inhibitor, and is a key constituent of standard ART regimens. Our group investigated the inhibition of HIV replication by AZT and its cytopathic effect by means of time-of-addition experiments using HIV-bearing MT-4 cells [15], confirming and extending previous work [16].

Given the history of marine organism-based drug discovery, attention subsequently turned to marine sponges, which proved to be a rich source of compounds with antiviral properties (Table 2). Cytarabine (Ara-C) is a structural analog of cytosine arabinoside and is currently used in the routine treatment of patients with leukemia and lymphoma [13]. Avarol blocks the synthesis of glutamine transfer RNA (tRNA), which is crucial for a viral protein synthesis [17]. Manzamine A is an alkaloid with a diverse range of bioactivity, including anti-HIV activity [18]. Mycalamide A was found to inhibit HSV-1 replication by blocking viral protein synthesis [19]. Papuamide A inhibits viral entry into host cells independently of the CD4-gp120/HIV and C–C chemokine receptor type 5 (CCR5)-gp41/HIV interactions [20].

Compounds extracted from algae have activity against a wide range of viruses, including HIV and HSV [21]. For example, galactan sulfate, which is a polysaccharide isolated from the red seaweed *Agardhiella tenera*, shows potent HIV replication-inhibitory activity [22]. A citrate buffer extract of the marine red alga *Schizymenia pacifca* inhibited RT of avian myeloblastosis virus and Rauscher murine leukemia virus [23], and the main component of this “sea algal extract” (SAE) was characterized as a member of the λ-carrageenan family, being a sulfated polysaccharide composed of galactose (73%), sulfonate (20%), and 3,6-anhydrogalactose (0.65%) with a molecular weight of approximately 2000 kDa [24]. SAE was demonstrated to be a specific inhibitor of HIV RT and HIV replication in vitro, and its sulfate residues were hypothesized to play a key role in the inhibition. Various types of sulfated polysaccharides such as dextran sulfate have been reported as potent inhibitors of HIV infection by researchers from around the world, including our group [25,26,27,28,29,30,31,32,33,34]. However, these observations did not generate much interest because the antiviral actions of these compounds were considered to be largely nonspecific [35]. Subsequent studies revealed that the target of sulfated polysaccharides is the binding of gp120/HIV to the cell-surface protein CD4 on naive cells [36,37,38].

Various plant-derived natural products have also been reported as potential lead compounds for anti-HIV agents. Lignin extracted from pine cones is a natural polyphenolic material generated by oxidative polymerization of phenylpropanoid monomers. Sakagami and co-workers have reported on the anti-HIV activity of lignin toward cultured cells [39,40]. Lignin was suggested to suppress the absorption of HIV onto the surface of cultured cells, although the details are unclear. Mitsuhashi et al. proposed that low-molecular-weight lignin inhibits HIV replication through suppression of HIV transcription from long terminal repeats (LTRs), including activation via nuclear factor kappa B (NF-κB) [41]. Betulinic acid (triterpenoid), isolated from the leaves of *Syzigium claviflorum*, inhibited HIV replication in a mechanism-blind screening [42]. The activity-directed derivatization of betulinic acid contributed to the creation of bevirimat, which disrupts core condensation by targeting a late step in Gag/HIV processing [43,44,45].

**Figure 4:**
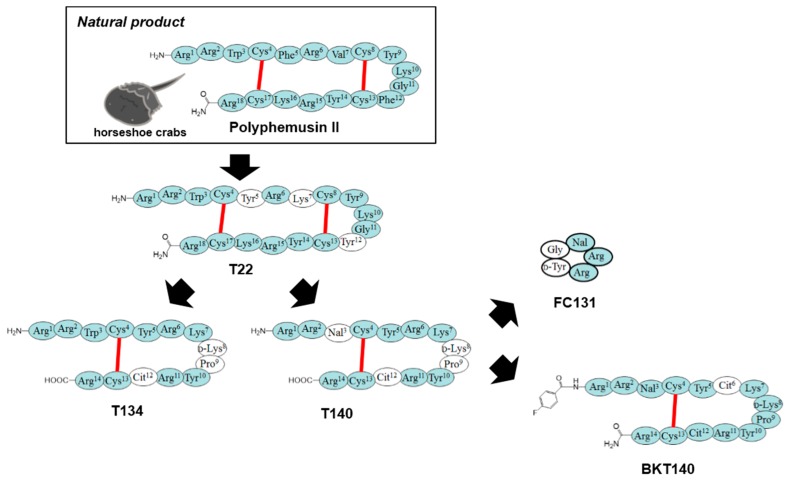
Discovery and development of C–X–C chemokine receptor type 4 (CXCR4) antagonists with potent anti-HIV activity from host-defense peptide of horseshoe crabs. Substitutions are highlighted in white. Red lines indicate intramolecular disulfide bond formation. Cit, l-citrulline; Nal, l-3-(2-naphthyl)alanine.

Cationic host-defense peptides, tachyplesins and polyphemusins, which were isolated from the hemocytes of horseshoe crabs (*Tachypleus tridentatus* and *Limulus polyphemus*), were reported to possess anti-HIV activity by Iwanaga’s group [46,47]. These peptides consist of 17 or 18 amino-acid residues with two intramolecular disulfide bridges. We investigated the structure–anti-HIV activity relationship (SAR) of these peptides and found that [Tyr-5,12, Lys-7]polyphemusin II (named T22) showed strong anti-HIV activity and low cytotoxicity [48,49]. Our continuing studies led to shortened polyphemusin analogs comprising 14 amino-acid residues, T134 [50] and T140 [51], as potent anti-HIV peptides. Moreover, FC131, which has a lower molecular weight than T134/T140, was found in a library of cyclic pentapeptides designed by means of a pharmacophore-guided approach based on SAR studies [52]. These peptides include a potent C–X–C chemokine receptor type 4 (CXCR4; HIV co-receptor) antagonist that strongly blocks X4-HIV-1 entry through competitive binding to CXCR4 [53,54,55]. Development of polyphemusin-derived CXCR4 antagonists from natural sources is a good example of a success story from natural product screening supported by fine synthetic technology using peptide chemistry (Figure 4). Currently, the biostable T140 analog BKT-140 (BioLineRx Ltd.) is a phase II drug candidate for the treatment of acute myeloid leukemia (AML) [56].

### 3.3. Antivirals in the 21st century

#### 3.3.1. Status of Anti-HIV Natural Products

The mainstream of antiviral discovery from natural sources continues to be directed against HIV and HSV. In the past decade, some unique compounds with anti-HIV activity have been reported from sponges, algae, and also from natural product libraries. Ma et al. reported the isolation of phenylspirodrimane, named stachybotrin D, from the marine sponge-associated fungus *Stachybotrys chartarum* MXH-X73, as a novel NNRTI [57]. Vidal et al. reported that daphnane diterpenes (daphnetoxin) extracted from the aerial parts of *Daphne gnidium* L. (Thymelaeaceae) with dichloromethane possessed anti-HIV inhibitory activity, interfering directly with the expression of the two main HIV co-receptors, CCR5 and CXCR4 [58]. Ixoratannin A-2 (doubly linked, A-type proanthrocyanidin trimer) and boldine (aporphine alkaloid) were identified as novel viral protein U (Vpu)/HIV-interacting anti-HIV inhibitors from the pan-African Natural Product Library (p-ANAPL), which is the largest collection of medicinal plant-derived pure compounds on the African continent [59]. The activities of these compounds require further characterization. The red algal protein griffithsin (GRFT) comprising 121 amino-acid residues shows promising potent anti-HIV inhibitory activity without cellular toxicity [60]. The potent HIV entry inhibitor GRFT was found to be a lectin that targets high-mannose *N*-linked glycans displayed on the surface of HIV envelope glycoproteins, and is of interest because unique technology has been developed for its large-scale production by genetic engineering using *Nicotiana benthamiana* plants transduced with a tobacco mosaic virus (TMV)-based vector expressing GRFT [61]. Recently, gnidimacrin, a daphnane-type diterpenoid extracted from the roots of *Stellera chamaejasme* (Thymelaeaceae), was reported to reduce latent HIV-1 DNA and the frequency of HIV-1-infected cells through activation of protein kinase C beta 1 and 2 (PKCβI and βII) in peripheral blood mononuclear cells (PBMC) from patients [62]. Persistent HIV infection is currently incurable owing to the presence of latent viral reservoirs of long-lived memory T cells, so targeting of latent viruses is an attractive strategy for complete HIV eradication, especially for ART-interrupted patients.

#### 3.3.2. Status of Anti-HSV Natural Products

The second most advanced antiviral development program after anti-HIV is anti-HSV. Anti-herpes drugs were the first antivirals targeting human pathogenic viruses and, thus, HSV infections are considered to be manageable, even though the available drugs have only limited therapeutic efficacy. It should be noted that chronic HSV infections of HIV-positive individuals, or solid organ transplant recipients, or patients with cancer may require prolonged antiviral treatment. In particular, HSV encephalitis is highly lethal. Unfortunately, prolonged therapies with available anti-herpes drugs may result in undesirable side effects, and can also induce the emergence of drug-resistant strains. Therefore, the discovery of novel anti-herpesvirus agents is still necessary.

There are many reports of compounds derived from various plant species as potential antiherpetic agents. Hassan et al. recently published a comprehensive review about bioactive natural products with anti-HSV properties, including nucleosides, polysaccharides, proteins, peptides, terpenes, phenolic compounds, and alkaloids [63]. Here, we focus on some of the materials currently considered most promising. Based on the successful development of ACV and Ara-A, marine organisms are anticipated to be a key source of novel anti-HSV drugs [64]. Mandal et al. showed that the polysaccharide xylan isolated from red algae *Scinaia hatei* exhibits activity against HSVs [65]. In addition, some sulfated polysaccharides, such as sulfated galactan, were reported to have antiviral activity for HSV [66,67], as well as HIV. These compounds may inhibit virus adsorption on cells. In 2005, a methanol extract of algae *Sargassum latiuscula* (Rhodomelaceae) collected in Korea was reported to display antiviral activities against not only wild-type HSV-1, but also ACV-resistant and/or thymidine kinase-deficient HSV-1 strains in vitro without apparent cytotoxicity, and it was also effective in a mouse HSV-1 infection model without noticeable toxic effects [68]. Fractionation of this extract afforded the active components 2,3,6-tribromo-4,5-dihydroxybenzyl methyl ether (TDB) and TDB alcohol. Meliacine isolated from *Melia azedarach* is a plant-derived glycopeptide exhibiting a therapeutic effect on HSV-induced ocular disease and genital herpetic infection in mice, possibly by inhibiting viral protein synthesis [69,70]. Various other natural products have yielded candidate anti-HSVs, including diterpenes from *Scoparia dulcis* L. (a medicinal plant) [71], a sulfated polysaccharide called calcium spirulan (Ca-SP) from *Spirulina platensis* (cyanobacteria) [72], fucoidan from *Undaria pinnatifida* (edible seaweed) [73], and nostoflan from *Nostoc flagelliforme* (terrestrial cyanobacterium) [74].

#### 3.3.3. Status of Natural Products with Activity against Other Viruses

Although HIV and HSVs have been the mainstream of attention, screening for and testing of natural compounds for activity against other viruses has also been progressing. For example, resveratrol (trans-3,4′,5 trihydroxystilbene) has in vitro antiviral activities against several members of the HHV family, including varicella-zoster virus (VZV) [75], EBV [76], human cytomegalovirus (HCMV) [77], and KSHV [78]. The compound appears to act on cellular pathways that affect viral replication, though the details are unclear. Anti-EBV peptide, an *N*-myristoylated peptide containing six amino acids, was isolated from hemolymph (blood) of larvae of tobacco budworm, *Heliothis virescens* [79]. This peptide has antiviral activity against several viruses, and Ourth proposed that the “myristate plus basic” motif in this peptide may prevent assembly and/or budding of viruses from the host cell. These two compounds are of interest, because there are no effective antiviral drugs or vaccines in clinical use for diseases caused by EBV and KSHV. Curcumin, a natural polyphenol derived from the rhizome of the medical plant *Curcuma longa Linn,* was reported to have anti-HPV activity due to downregulation of HPV18 transcription via inhibition of activator protein 1 (AP-1) [80]. This seems noteworthy, because curcumin is readily available and inexpensive. Slater et al. reported that indolocarbazoles derived from the natural product arcyriafavin A (an alkaloid) are potent and selective inhibitors of replication of HCMV [81]. Arcyriafavin A is a potent cyclin-dependent kinase 4 (CDK4)/cyclin D1 and Ca^2+^/calmodulin-dependent (CaM) kinase II inhibitor, and seems to be a promising candidate for further development.

At present, the most promising next-generation antiviral agents from natural sources may be the red algal protein GRFT and the algae-derived polysaccharide carrageenan (CG). GRFT is a potent anti-HIV agent, and also inhibits infection with other sexually transmitted infectious viruses, including HSV by targeting viral entry and cell-to-cell transmission [82], HPV by mediating receptor internalization [83], and HCV by targeting cell entry [84]. CG blocks the binding of HPV to cells [85,86], and is well established as a thickening agent in various foods and cosmetic products, including some brands of sexual lubricant [87]. It has, therefore, the advantage of being recognized as safe by the Food and Drug Administration (FDA), and a microbicide gel formulation for vaginal application has been developed, taking advantage of its gel-forming property. Recently, the combination of GRFT and CG was reported to show broad antiviral activity against HSV-2 and HPV in murine models [83]. The combination of GRFT and CG seems promising, and we discuss it further below in connection with new pharmaceutical formulation technologies.

## 4. Formulation of Natural-Product-Derived Antivirals Using New Pharmaceutical Technologies

Recently, innovations in drug formulation technology have attracted great interest as a means of improving clinical outcomes. Drug delivery systems (DDS) such as nano- and microparticles, targeted carriers, prodrugs, polyethylene glycol (PEG)ylation, hydrogel depots, and so on are attractive technologies to enable a drug to act at the right time, at the right site, and at the required concentration. The objectives of these systems include controlled drug release, prolongation of drug lifetime, acceleration of drug permeation and absorption, and drug targeting. For example, a prodrug modification of ACV is the l-valyl ester (VACV), which shows increased cell-membrane permeability, enabling a reduction in the frequency of administration and reduced side effects. Some formulations of natural products themselves have been reported to serve as topical microbicides. Cellulose sulfate, an HIV entry inhibitor in vitro, was investigated for use as a sulfated polysaccharide vaginal gel formulation [88,89]. Unfortunately, however, no significant effect of cellulose sulfate on the risk of HIV acquisition was found, compared with the placebo [90]. In an alternative approach, sodium carboxymethylcellulose (Na CMC) has been used as a gelling agent together with maraviroc and tenofovir (both of which are in clinical use) for prevention of rectal acquisition of HIV [4]. In a macaque model, these drugs were detectable in plasma at 30 minutes after gel application and remained in rectal fluids at more than 95%-inhibitory concentrations for 24 h. The algae-derived anti-HPV polysaccharide CG (see Section 3.3.3) is a macromolecular gel, and the CG-based gel formulation Carraguard is now a phase III drug candidate as a sexual lubricant with anti-HPV properties. More recently, the combination of the red algal protein GRFT and CG in a novel formulation called a freeze-dried fast-dissolving insert (FDI) was reported to protect rhesus macaques from vaginal simian–human immunodeficiency virus (SHIV) challenge, as well as mice from vaginal HSV-2 and HPV pseudovirus challenge, and current phase I trials are looking at the anti-HPV properties of a GRFT/CG combination gel as a sexual lubricant [91]. Thus, formulations with good retention at invasion sites using natural products acting outside infected cells, e.g., entry and/or budding inhibitors for host cells, seem promising, especially for pre-exposure prophylaxis (PrEP).

Some drug formulation studies of curcumin, an anti-HPV natural product from medical plants, were recently reported. Curcumin inspired considerable interest based on its extensive physiological activities; however, poor bioavailability restricts its clinical translation. Treatment of cervical cancer with a curcumin nanoparticle formulation in poly(lactic-*co*-glycolic acid) (PGLA) was investigated in an orthotopic mouse model [92,93]. Various hydrogel formulations for curcumin have also been proposed [94,95]. The results of these pre-clinical experiments suggested that suitably formulated curcumin could be an effective therapeutic modality for HPV-induced cervical cancer.

## 5. Prospects

Most viral infections of the oral cavity involve HHVs, especially in HIV-infected individuals. As described in Section 2, HSV type 1 (HHV-1) and 2 (HHV-2) produce shallow, small, painful ulcers which may coalesce. The development of antiviral agents with their delivery systems that successfully stop the recurrence of oral ulceration (recurrent aphthous stomatitis (RAS)) is anticipated [96]. VZV (HHV-3) is responsible for chickenpox upon primary infection, and shingles in its reactivated form. EBV (HHV-4) causes infectious mononucleosis and/or glandular fever. HCMV (HHV-5) can cause large, painful ulceration on any oral surface. EBV and HCMV were also reported to associate with periodontitis [97]; therefore, the discovery of new antiviral agents is necessary, especially for chronic periodontitis. HHV-6 and -7 are associated with facial rashes in babies and oral ulceration. Also, reactivated HHV-6 can cause encephalitis in patients after transplantation [98]. KSHV (HHV-8) is associated with KS in AIDS patients. In HIV-infected individuals, herpes infections often persist for long period of time. In addition, HPV infections are also found in AIDS patients, and may give rise to exophytic warts, often at the corners of the mouth. In patients with AIDS, there is a danger that viremias may spread to life-threatening sites, and early treatment of oral herpetic infections is essential.

Recent developments in anti-HIV drugs has mainly been focused on creating novel formulations to improve medication compliance, such as mixed formulations including drugs with different mechanisms of action in a single tablet. For example, Complera^®^ is a once-a-day medication, consisting of a single tablet containing rilpivirine (NNRTI), emtricitabine (NRTI), and tenofovir (NRTI). Prezcobix^®^ utilizes a pharmacokinetic (PK) booster strategy, with a single tablet containing darunavir (PI) and a cytochrome P450 3A (CYP3A) inhibitor to prolong the blood half-life of darunavir. We have reported a depot strategy for the peptide drug Fuzeon^®^, which is used for the treatment of HIV-infected individuals and AIDS patients with multidrug-resistant HIV infections [99]. Natural-product-derived peptides with anti-viral activity may be candidates for PrEP and post-exposure therapy if appropriate pharmaceutical technology is employed. Also, there is still an enormous range of natural resources (plants, etc.) that remain to be explored for new antiviral candidates. Thus, discovery and development of new drugs in combination with improved pharmaceutical formulation technologies offers great promise for the future treatment of viral infections.

## Figures and Tables

**Table 1:** Human herpesviruses (HHVs) and their associated diseases.

Type	Target	Oral Manifestations	Other Pathology
1. HSV-1	Mucoepithelial	Herpes ulcers	Genital ulcers
2. HSV-2	Mucoepithelial	Herpes ulcers	Genital ulcers
3. VZV	Mucoepithelial	Possible oral manifestations of chicken pox and herpes zoster	Chicken pox and herpes zoster
4. EVB	B cells and epithelial cells	Hairy leukoplakia, Periodontitis (nasopharyngeal carcinoma)	Mononucleosis and lymphoma
5. HCMV	Monocytes, lymphocytes, and epithelial cells	Periodontitis	
6. HHV-6	Monocytes and macrophages		Roseola in infants
7. HHV-7	T cells and possibly others		Roseola in infants
8. KSHV	B cells and possibly others		Kaposi sarcoma (in AIDS patients)

HSV-1, herpes simplex virus 1; HSV-2, herpes simplex virus 2; VZV, varicella-zoster virus; EBV, Epstein–Barr virus; HCMV, human cytomegalovirus; HHV-6, human herpesvirus 6; HHV-7, human herpesvirus 7; KSHV, Kaposi sarcoma-associated herpesvirus; AIDS, acquired immune deficiency syndrome.

**Table 2:** Sponge-derived antivirals reported in the latter part of the twentieth century. Compounds below the horizontal dotted line are more recent medicines. HIV—human immunodeficiency virus.

Compound	Organism	Target Virus	Reference
Acyclovir	*Tethya cripta*	HSV, VZV	Elion et al., 1977 [12]
Cytarabine	*Tethya cripta*	HSV *^1^	Privat and de Rudder, 1964 [13]
Vidarabine	*Tethya cripta*	HSV	Privat and de Rudder, 1964 [13]
Zidovudine	*Tethya cripta*	HIV	Horwitz et al., 1964 [14]
Avarol	*Disidea avara*	HIV	Muller et al., 1987 [17]
Manzamine A	*Haliclona* sp.	HIV	Sakai et al., 1986 [18]
Mycalamide A	*Mycale*	HSV	Perry et al., 1988 [19]
Papuamide A	*Theonella mirabilis*		Ford et al., 1999 [20]

*^1^: presently used as an anti-tumor agent.
